# Artemisitene shows superiority over artemisinin in preventing* Schistosoma japonica*-induced liver disease

**DOI:** 10.1186/s13071-024-06426-y

**Published:** 2024-08-15

**Authors:** Meng-Ke Liu, Xu-Yang Chen, Juan-Juan Tang, Zhi-Peng Liu, Gui-Ying Lin, Jun-Ling Cai, Zuo-Ming Chen, Yu-Yun Yan, Xiao-Fang Ji, Zhong-Jin Yang, Zi Li

**Affiliations:** 1 Sino-French Hoffmann Institute, School of Basic Medical Sciences, Guangzhou Medical University, Guangzhou, 511436 China; 2 School of Chemistry and Chemical Engineering, Tianjin University of Technology, Tianjin, 300384 China; 3 The Second Affiliated Hospital of Guangzhou Medical University, State Key Laboratory of Respiratory Disease, Guangdong Provincial Key Laboratory of Allergy and Clinical Immunology, Guangzhou Medical University, Guangzhou, 510260 China

**Keywords:** Artemisinin, Artemisitene, *Schistosoma japonica*, Liver fibrosis, Tegument, Host immunity

## Abstract

**Background:**

Artemisinin (ART) analogs, such as dihydroartemisinin, arteether, artemether, and artesunate, all featuring an endoperoxide bridge, have demonstrated efficacy against schistosomiasis. Artemisitene (ATT), which contains an additional α, β-unsaturated carbonyl structure, has shown enhanced biological activities. This study aims to evaluate the anti-schistosomaiasis japonica activity of ATT and compare it with ART.

**Methods:**

We assessed liver inflammation and fibrosis in mice using hematoxylin and eosin staining and Sirius red staining, respectively. RNA sequencing analyzed transcriptomics in female and male *Schistosoma japonicum* (*S. japonicum*) adult worms and mice livers, with cytokine profiling and flow cytometry to study immune responses under ART or ATT treatment.

**Results:**

ATT exhibits a marked reduction in female *S. japonicum* adult worms and egg numbers, damaging the adult worms’ surface. It also influences the transcription of genes related to cellular anatomical structures. Notably, ATT treatment resulted in significant reductions in liver granuloma size and collagen area, alongside lowering serum levels of glutamic pyruvic and glutamic oxaloacetic transaminase more effectively than ART. Both ART and ATT markedly decreased neutrophil frequency in the liver and elevated eosinophil counts. However, only ATT treatment significantly reduced the M1/M2 and Th1/Th2 indices, indicating a pronounced shift in immune response profiles. ATT-affected host immunity correlated with the extent of liver fibrosis and the count of single males more strongly than ART.

**Conclusion:**

ATT, as a novel preventive strategy for schistosomiasis japonica in mice, significantly outperforms ART.

**Graphical Abstract:**

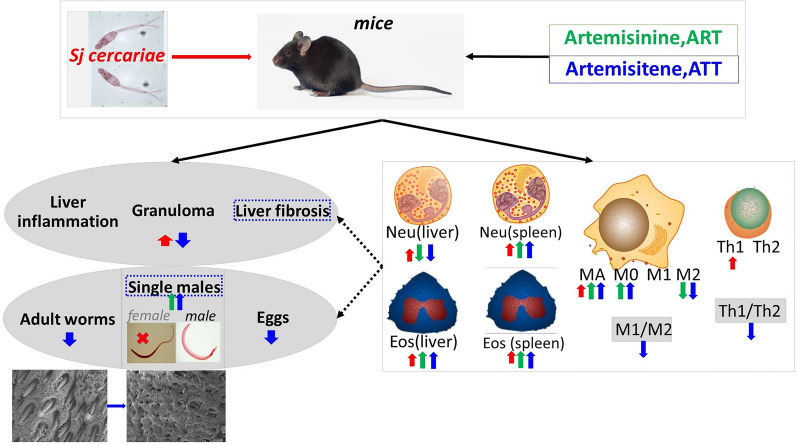

## Background

Schistosomiasis, a helminthic disease caused by blood flukes, affects over 300 million people and causes approximately 11,000 deaths annually. According to the World Health Organization (WHO) 2023 data, schistosomiasis is endemic in 78 countries across Asia, Africa, the Middle East, South America, and the Caribbean, with 51 of these countries requiring preventive drug administration due to moderate to severe transmission rates [1–4]. *Schistosoma japonicum* is the main causative agent in Asia, affecting countries such as the Philippines, China, and Indonesia. As a One Health concern, *S. japonicum* can infect not only humans but also more than 40 species of wild and domestic animals, including dogs, pigs, rats, goats, sheep, and bovines. The broad host range and transmission through freshwater snails add complexity to control strategies [4, 5]. The pathogenic life cycle of *S. japonicum* begins with cercariae, which are released from snails and penetrate the skin of the host. This leads to the formation of *schistosomula* in the skin and subsequently in the lungs. After about 5 weeks of infection, adult male and female worms mature and release a large number of eggs. The eggs and secretions from adult worms living in the hepatic portal vein trigger host immune responses, leading to liver granulomatous inflammation and fibrosis. By 6 weeks, the host exhibited acute schistosomiasis japonica, characterized by serious granulomatous inflammation but relatively mild fibrosis [4, 6–8].

The development of hepatic pathology in acute liver schistosomiasis japonica is significantly influenced by the concerted actions of innate and adaptive immune cells. This includes neutrophils, macrophages, eosinophils, and T helper cells (Th), along with the various cytokines and chemokines they produce. Neutrophils, in particular, induce the formation of granulomas and liver fibrosis [9, 10]. Studies in mice have shown that interferon-γ (IFN-γ) is crucial for recruiting neutrophils and enhancing the antipathogen response through the release of neutrophil extracellular traps (NETs) during the granulomatous response [6, 7]. Interestingly, *S. japonicum* has been observed to hinder NET formation in wild-type mice by upregulating interleukin-10 (IL-10) expression [10]. In contrast, during *Schistosoma mansoni* infections in mice, eosinophils, though known for their capabilities in clearing parasites, are not essential for the development of granulomas or liver fibrosis. However, they are also a key component of granulomas and serve as an important source of IL-4, which is essential for sustaining Th2 responses [11, 12]. Macrophages, as part of the mononuclear phagocytic system, are implicated in tissue homeostasis and various infections. They are categorized into two main subtypes: the classically activated macrophage (M1), which exhibits pro-inflammatory actions, and the alternatively activated macrophage (M2), known for its anti-inflammatory activity. The M1/M2 paradigm mirrors the Th profiles, Th1 and Th2. Notably, macrophages are the most abundant cells in the liver granulomas of mice infected with *S. mansoni* [6]. During the acute phase of a *Schistosoma* infection, generally occurring around 5–7 weeks post-infection, the immune response is predominantly Th1 type. This phase is marked by an increased number of M1 macrophages, which are known for producing interleukin-12 (IL-12), interleukin-6 (IL-6), tumor necrosis factor-alpha (TNF-α), and nitrogen oxide (NO) [9, 13]. Interestingly, the sixth week of *S. japonicum* infection induces a moderate Th2 response, which is less pronounced than that observed in the seventh week of infection [6, 14]. An excessive Th1 response can lead to severe acute cachexia and potentially death, whereas Th2 immunity acts as a double-edged sword. On one hand, Th2 immunity offers anti-inflammatory effects and suppresses Th1-mediated immunopathological changes. On the other hand, it can drive liver inflammation and even liver fibrosis. Therefore, maintaining a balance between Th1 and Th2 responses is critical for the effective control of schistosomiasis.

Praziquantel (PZQ) is currently the only drug used in the clinical treatment of schistosomiasis. The WHO recommends mass drug administration (MDA) of PZQ, primarily targeting school-aged children, to better control and eliminate the disease [15, 16]. This need arises from its limited effectiveness against juvenile worms. Consequently, there is a pressing demand for novel therapeutic agents. Artemisinin (ART)-based combination therapies are the first-line treatment for uncomplicated malaria infection [17, 18]. However, ART-resistant strains of *Plasmodium falciparum* have emerged in the Greater Mekong region and subsequently in Africa [19]. In experimental models of schistosomiasis and clinical trials, ART derivatives such as dihydroartemisinin, arteether, artemether, and artesunate, all featuring a distinctive endoperoxide bridge, have demonstrated efficacy against juvenile *Schistosoma* worms [20–23]. Additionally, artemisinin-type compounds have demonstrated a regulatory effect on inflammation and cell type- and context-dependent innate and adaptive immune responses, further highlighting their potential in schistosomiasis prevention and treatment strategies. However, in vivo animal studies showed the possibility of neurotoxicity for both artemisinin and these analogs. Therefore, new artemisinin derivatives with high efficiency are required. Artemisitene (ATT) retains the endoperoxide moiety and contains α, β-unsaturated carbonyl structure, which inhibits the expression of inducible NO oxidase (iNOS) and cyclooxygenase-2 (COX-2), potentially enhancing some biological activities. The effect of ATT on parasitic diseases remains unclear.

Considering the schistosomicidal potential of ATT, we investigated its anti-schistosomiasis japonica activity in this study, which had not been previously tested. We also tested ART to allow a direct comparison between ATT and ART, as their differing chemical structures might result in varying efficacies. Furthermore, we examined and compared their effects on host immunity to deduce the possible mechanisms involved.

## Methods

### Reagents

ART was purchased from MedChemExpress (HY-B0094), and ATT was produced and identified the research group led by Professor Zhong-jin Yang at the College of Pharmacy, Guangzhou Medical University [24]. Alanine transaminase (ALT) microplate assay kit (abs580004) and aspartate transaminase (AST) microplate assay kit (abs580002) were purchased from Absin Biotechnology Co Ltd (Shanghai, China). Hematoxylin and eosin (H&E) staining kit (BA-4025, Baso Biological Technology Co., Ltd, Zhuhai, China), Bio-Plex Pro-Mouse Group I Cytokine 23-plex assay (M60009RDPD, Bio-Rad, Hercules, California, USA), and Sirius red staining kit (ab150681, Abcam, Cambridge, UK) were ordered. Antibodies against mouse alpha-smooth muscle aorta (α-SMA) from Abcam (ab124964), CD45-PE-Cyanine 7 (30-F11, cat. no. 25-0451-82), CD206-Alexia Flur™ 488 (MR6F3, cat. no. 53-2061-82), the inducible isoform of nitric oxide synthase (iNOS)-APC-eFlur 780 (CXNFT, cat. no. 47-5920-82), IL-4-PerCP-eFlur 710 (11B11, cat. no. 46-7041-82), IFN-γ-PE-Cyanine 7 (XMG1.2, cat. no. 25-7311-82), F4/80-PE (T45-2342, cat. no. 565410), fixable viability stain—BV510 (cat. no. 65-0866-14), CD4-BV421 (RM4-5, cat. no. 100531), CD3-FITC (145-2C11, cat. no. 100305), and each of their corresponding isotype controls were obtained from eBioscience (San Diego, CA, USA). Anti-mouse-CD11b-PerCP-Cy^TM^5.5 (M1/70, cat. no. 550993), SiglecF-Alexa Fluor 647 (E50-2440, cat. no. 562680), and Ly6G-BV421 (IA8, cat. no. 562737) were from BD Biosciences (USA).

### Mice, parasite infection, and ART or ATT treatment

**Fig. 1:**
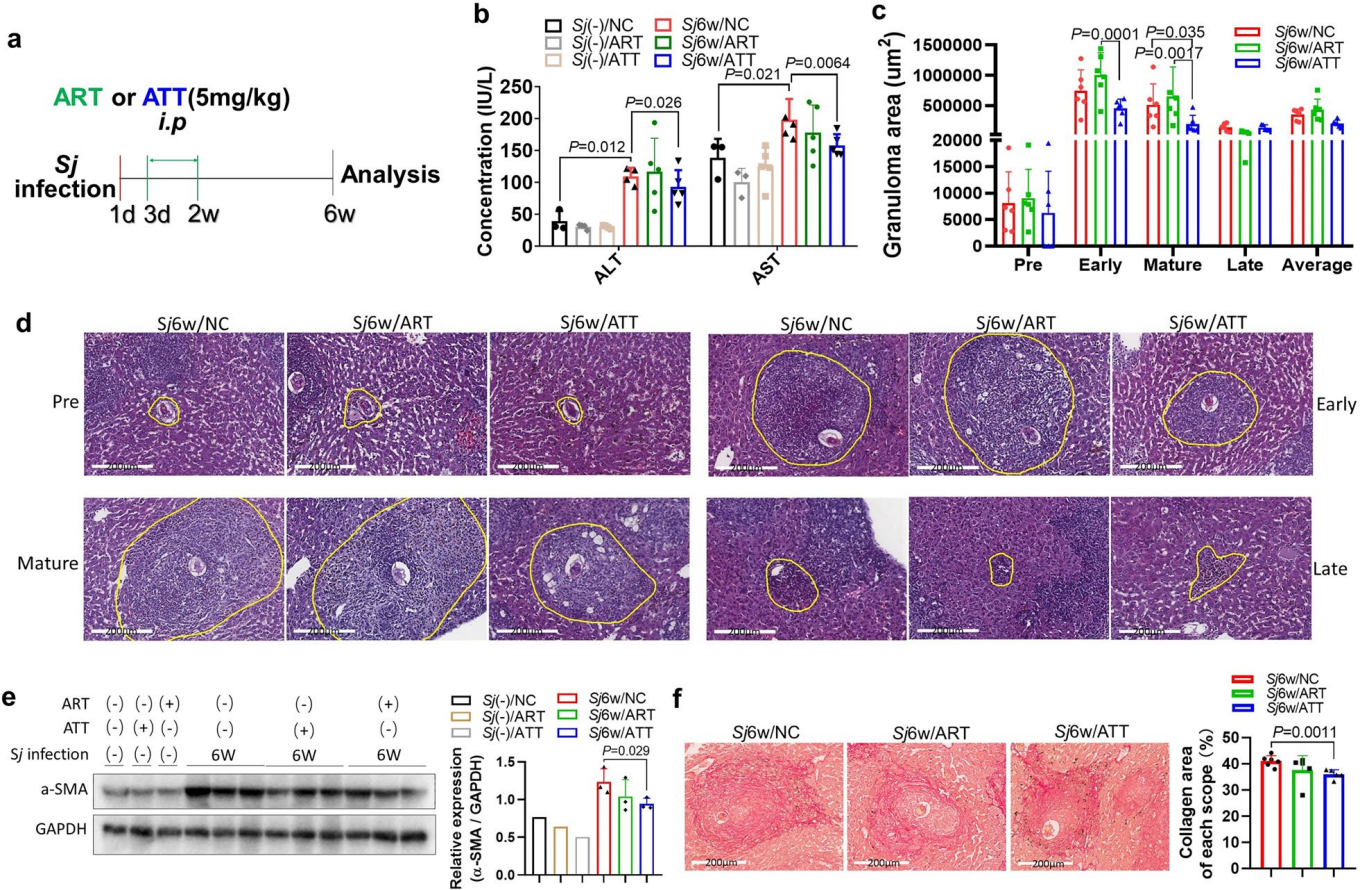
Differential effect of ATT and ART on *S. japonicum* infection‑induced liver inflammation and fibrosis in mice. **a** Experimental flowchart depicting the treatment of mice infected with *S. japonicum* cercariae. After a 6-week infection period, mice received intraperitoneal injection of ART or ATT (5 mg/kg body weight) from day 3 to 14 post-infection. **b** Serum levels of ALT and AST were measured and analyzed in the indicated mouse groups using a *t*-test (*n* = 4 for uninfected; *n* = 5 or 6 for infected). **c**, **d** Liver sections were stained with H&E. The granuloma area percentages, encompassing pre-, early, mature, and late stages around a single egg in each liver section, were quantified using ImageJ and analyzed by two-way ANOVA (**c**). Representative images of granulomas at different stages around a single egg from each mouse group are presented (**d**). **e** α-SMA levels in mouse liver tissues were assessed by western blotting (WB) (left panel), and semiquantitative analysis of this protein was performed using ImageJ software (right panel, *t*-test). **f** Liver sections were stained with Sirius red (200 μm) to visualize *S. japonicum* egg granulomas. Images show representative staining from mice with and without ART or ATT treatment (left panel). The morphometric analysis of collagen areas around a single egg (stained in strong red) was conducted, and the results are presented (right panel, *t*-test). Data represent mean ± SD from different group experiments. Significant differences are indicated as *P < 0.05 and **P < 0.01

Female C57BL/6 mice, aged between 6 and 8 weeks, were sourced from the Specific Pathogen-Free (SPF) Guangdong Medical Laboratory Animal Center. These mice were maintained in accordance with institutional guidelines. The mice were infected with approximately 20 ± 3 *S. japonicum* cercariae of the Chinese mainland strain via abdominal skin penetration. Treatment involved administering either ATT or ART at a dosage of 5 mg/kg body weight, dissolved in a mixture of 2% DMSO, 20% PEG300, and 78% saline. This treatment was given intraperitoneally once daily from day 3 to day 14 post-*S. japonicum* infection to each mouse in the treatment group (*n* = 6). The infection control group received only the solvent mixture (2% DMSO, 20% PEG300, and 78% saline, *n* = 6). Additionally, two noninfected control groups were established: one treated with ATT (*n* = 4) and the other with the solvent mixture (*n* = 4). At week 6 post-*S. japonicum* infection, the mice were anesthetized using inhalational ether and then sacrificed by cervical dislocation. Subsequently, sera, spleens, and liver tissues were collected for further analysis (Fig. 1a). All procedures involving these mice were approved by the Institutional Animal Care and Use Committee at South China Agricultural University and Guangzhou Medical University (approval no. 2022-115) and conformed to the Guidelines for the Care and Use of Laboratory Animals of the National Institute of Health in China.

### H&E staining

Fresh hepatic tissues were initially fixed in 4% paraformaldehyde for 24 h and then embedded in paraffin for preservation. Liver sections with a thickness of 4 µm were prepared and stained with hematoxylin and eosin (H&E). This staining was conducted to facilitate the counting of *S. japonicum* eggs and to assess both the size of granulomas and the extent of liver granulomatous inflammation. The severity of liver granulomatous inflammation was evaluated based on the criteria established in previous studies [6, 25].

### Scanning electron microscopy of *S. japonicum* adult worms

The ultrastructural features of both female and male adult *S. japonicum* worms from mice treated with ATT were meticulously examined using scanning electron microscopy (SEM). This analysis was compared with observations from worms in untreated mice. For SEM preparation, the worms were initially rinsed thrice in phosphate-buffered saline (PBS, pH 7.4) and subsequently fixed overnight at 4 °C in a 2.5% glutaraldehyde–PBS solution (pH 7.4). Then, the worms underwent another round of washing in PBS and then fixed in 1% osmium tetroxide (OsO4) solution for 1 h. The dehydration process involved a graded series of ethanol concentrations. Finally, worms were dried, affixed onto aluminum stubs, and sputter-coated with gold, after which they were examined with a Hitachi SU8100 SEM (Chiyodaku, Japan).

### Transcriptome analysis through RNA sequencing (RNA-seq)

Adult *S. japonicum* worms, either female or male, were carefully harvested from the portal and mesenteric veins, as well as liver tissues of mice infected with *S. japonicum*. These samples were collected from treated or untreated groups with ATT. The worms were thoroughly washed thrice with PBS to ensure cleanliness. Following this, Total RNA was extracted using TRIzol Reagent (Invitrogen), and its purity and concentration were assessed. Subsequently, the RNA samples were sequenced using the BGISEQ-500 platform provided by BGI Company in China. Differentially expressed genes (DEGs) were identified. The analysis to enrich functions and signaling pathways analysis were conducted utilizing the Gene Ontology (GO) and the Kyoto Encyclopedia of Genes and Genomes (KEGG) database.

### Sirius red staining

Paraffin-embedded liver tissues were cut into sections with 4 µm thickness. These sections were then stained with Sirius Red to highlight collagen areas, which appear in red. The extent of these red areas was semiquantitatively assessed using ImageJ software. To ensure unbiased evaluation, each stained sample was independently reviewed in a double-blind manner by two separate researchers.

### Western blotting (WB)

The liver tissues from the selected mice were processed using a radio-immunoprecipitation assay buffer (RIPA, P0013B, Beyotime, Shanghai, China). The protein concentrations of these samples were then quantified using a bicinchoninic acid assay (BCA) protein assay kit (Dingguo, Cat no BCA02). Equal quantities of total protein lysates were separate using 10% sodium dodecyl sulfate polyacrylamide gel electrophoresis (SDS–PAGE) and subsequently transferred onto polyvinylidene difluoride (PVDF) membranes. For protein detection, the membranes were incubated firstly with an anti-α-SMA (1:10,000) primary antibody, followed by a HRP-conjugated secondary anti-rabbit antibody. The α-SMA protein was visualized using an ECL Western Blotting Substrate (GBCBIO, cat. no. 03308) and captured with Fusion SoloS (FUSION-CAPT/EVOLUTION-CAPT). The intensity of α-SMA band from the mice liver samples was semi-quantitatively analyzed using ImageJ and normalized against the GAPDH band.

### Multi-cytokines detection

Liver lysate samples were prepared with RIPA lysis buffer (P0013B, Beyotime Institute of Biotechnology, Shanghai, China) and stored at − 80 ℃. Prior to analysis, these samples were allowed to equilibrate at room temperature for 30 min. The quantification of cytokines were conducted using Bio-Plex Pro-Mouse Group I Cytokine 23-plex assay kit (M60009RDPD, Bio-Rad, Hercules, California, USA) in conjunction with Bio-Plex^®^ 200 System, MAGPIX Multiplex Reader (Bio-Rad Laboratories, Life Science Group 2000, Hercules, CA, USA). This process employs the cutting-edge Luminex^®^ xMAP^®^ technology, an immunoassay method renowned its capability to simultaneously to quantify up to 23 different targets: IL-1α, IL-1β, IL-2, IL-3, IL-4, IL-5, IL-6, KC, IL-9, IL-10, IL-12(p40), IL-12(p70), IL-13, IL-17A, Eotaxin (CCL11), granulocyte colony-stimulating factor (G-CSF), granulocyte–macrophage colony-stimulating factor (GM-CSF), interferon-gamma (IFN-γ), monocyte chemoattractant protein 1 (MCP-1), macrophage inflammatory protein- (MIP)-1α and MIP-1β, regulated on activation normal T expressed and secreted (RANTES or CCL5), and tumor necrosis factor α (TNF-α). The concentration of each cytokine was accurately extrapolated from its specific calibration curve, tailored individually for each cytokine. This determination was independently conducted for each experiment, corresponding to each assay plate. Furthermore, the sample size from each group consisted of more than four specimens.

### Preparation of single‑cell suspensions of mice liver and spleen

Mice were gently anesthetized, followed by an infusion of sterile normal saline into the left ventricle, ensuring thoroughly removal of blood from organs. Then, the livers were excised, segregated using forceps, and digested with 0.25 mg/mL liberase (589994, Roche^®^ Life Science) and 0.5 mg/mL DNase I (Roche, 10104159001) for 1 h at 37 ℃. The digested liver tissues, along with the dissected spleen, were meticulously processed over a 100-µm cell strainer (BD Falcon) to obtain single-cell suspensions, which were then suspended in Hanks’ balanced salt solution (HBSS). Red blood cells were lysed and removed using ammonium chloride for 10 min. Subsequently, cell suspensions were incubated with LIVE/DEAD Zombie NIR™ Fixable Viability Kit (BioLegend) for 20 min, followed by resuspension at a concentration of 2–3 × 10^6^ cells/ml in complete Roswell Park Memorial Institute (RPMI) 1640 medium with 10% fetal bovine serum (FBS).

### Cell surface and intracellular cytokines staining and then flow cytometry analysis

The cell suspensions were initially pre-treated with mouse Fc block antibody (BD, clone 2.4G2) to minimize non-specific binding. A variety of antibodies were used for staining cell surface markers, including anti-CD45, CD11b, Ly6G, F4/80, SiglecF, CD206, and iNOS, as well as anti-CD3e, γδTCR, CD4. For intracellular staining of IL-4 or IFN-γ, the cells were stimulated using a combination of phorbol 12-myristate 13-acetate (20 ng/ml; Sigma-Aldrich), ionomycin (1 μg/ml; Sigma-Aldrich), and brefeldin A (BFA; 10 μg/ml; Sigma-Aldrich) for 4 h at 37 °C. Then, the cells were fixed, permeabilized, and stained. Flow cytometry analysis was conducted using the CytoFLEX system (Beckman Coulter) and data were analyzed with FlowJo software (Ashland, OR, USA).

Neutrophils were gated as CD45^+^CD11b^+^Ly6G^+^. Eosinophils were identified as CD45^+^CD11b^+^SiglecF^+^. Macrophages were categorized as CD45^+^Ly6G^−^F4/80^+^. Additionally, macrophage subtypes were differentiated as follows: M0 macrophages were identified as CD45^+^Ly6G^−^F4/80^+^iNOS^−^CD206^−^; M1 macrophages as CD45^+^Ly6G^−^F4/80^+^ iNOS^+^; and M2 macrophages as CD45^+^Ly6G^−^F4/80^+^CD206^+^. For T helper cell subtypes, Th1 cells were gated as CD3^+−^CD4^+^IFN-γ^+^, and Th2 cells as CD3^+^ CD4^+^IL-4^+^.

### Statistical analysis

The results are presented as the standard deviation (± SD) based on the number of specified replicates or experiments. Data from each group were analyzed using Prism 8.0. The statistical evaluation of differences between any two groups was performed using a two-tailed Student’s *t*-test. For the comparison of multiple groups, one-way or two-way analyses of variance (ANOVA) were conducted. Correlation analyses were performed by OriginPro 2021’s Correlation Plot. A *P* value of 0.05 or lower was considered indicative of statistical significance.

## Results

### ATT treatment, as opposed to ART, significantly reduced the severity of liver inflammation and fibrosis induced by *S. japonicum* infection

Anti-inflammatory and antifibrotic effects have been observed in various ART analogs. Mice infected with *S. japonicum* for 6 weeks exhibited pronounced liver inflammation and fibrosis. Our findings reveal that 6 weeks of *S. japonicum* infection markedly increased the concentration of sera ALT and AST, both indicators of liver inflammation or damage [26]. Importantly, treatment with ATT, but not ART, significantly decreased these levels (Fig. 1b) (*t*-test: *Sj*(−)/NC versus *Sj*6w/NC: ALT: *t*(5) = 6.37, *P* = 0.012; AST: *t*(6) = 2.56, *P* = 0.021. *Sj*6w/NC versus *Sj*6w/ATT: ALT: *t*(7) = 1.15, *P* = 0.026; AST: *t*(8) = 2.41, *P* = 0.0064). Furthermore, *S. japonicum* eggs-induced liver granulomas, which can be classified into pre, early, mature, and late stages [6, 7], were substantially reduced by ATT treatment alone. This reduction was particularly notable in granulomas at their early and mature stages, as evidenced by H&E staining (Fig. 1c and d; ANOVA: *F*(8,73) = 2.27, *P* = 0.032. *Sj*6w/NC versus *Sj*6w/ATT: mature: *P* = 0.035; *Sj*6w/ART versus *Sj*6w/ATT: early: *P* = 0.001, mature: *P* = 0.0017).

The primary markers for activated hepatic stellate cells (HSCs) and myofibroblasts in mice are α-SMA and collagen secretion. Notably, ATT treatment significantly lowered the expression of α-SMA, as evidenced by WB analysis (Fig. 1e; *t*-test: *Sj*6w/NC versus *Sj*6w/ATT: *t*(4) = 2.63, *P* = 0.029). Additionally, ATT significantly alleviated the area of collagen deposition surrounding individual eggs, as demonstrated by Sirius red staining of liver sections (Fig. 1f; *t*-test: *Sj*6w/NC versus *Sj*6w/ATT: *t*(9) = 4.26, *P* = 0.0011). These findings suggest that ATT is more effective than ART in ameliorating liver fibrosis induced by *S. japonicum* infection in mice.

### ATT demonstrated a potent schistosomicidal activity in mice, surpassing the effect of ART

**Fig. 2:**
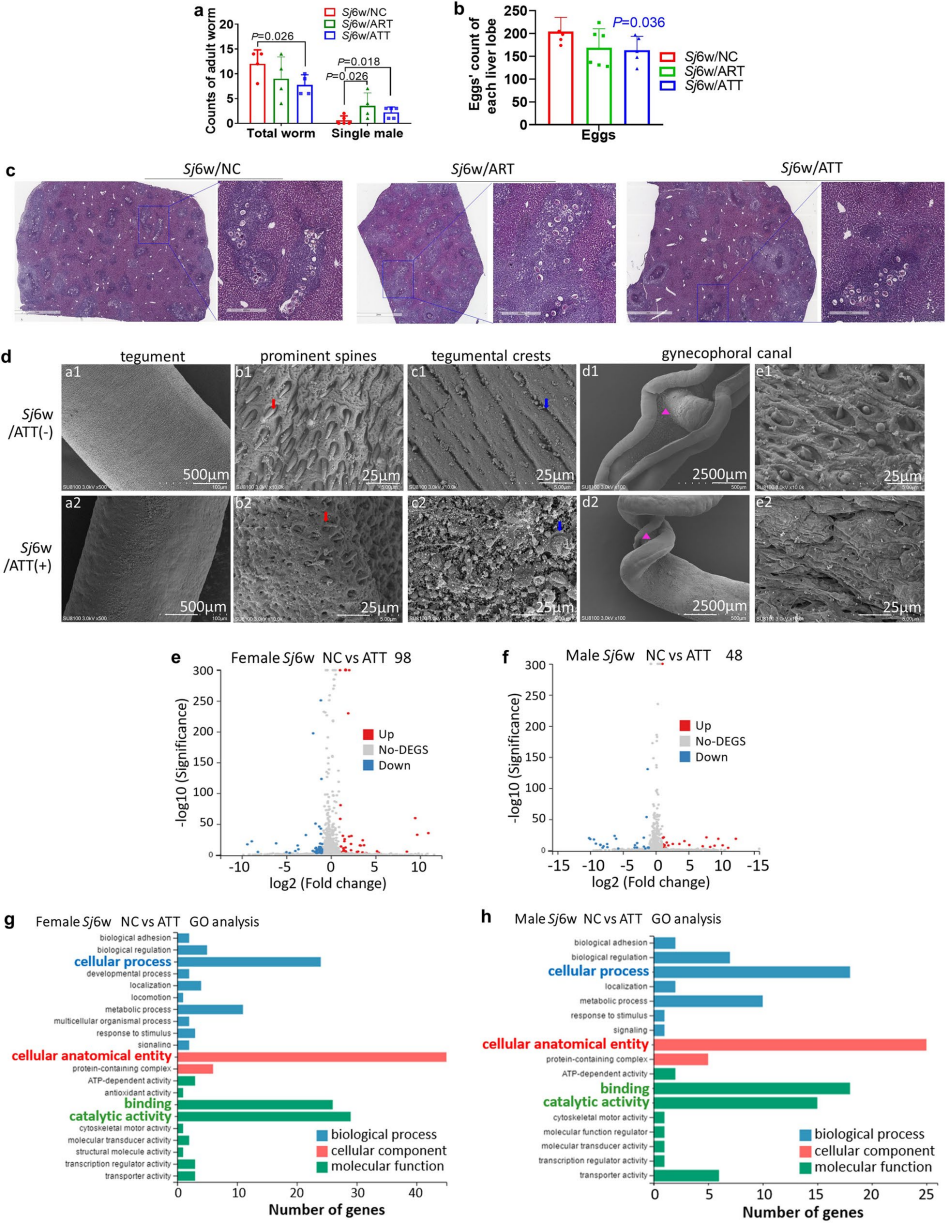
Enhanced anti-*S. japonicum* activity of ATT compared with ART in mice. **a** The total number of *S. japonicum* adult worms and individual male worms present in the hepatoportal and mesenteric veins of mice with 6 weeks of *S. japonicum* infection, with either ART or ATT treatment (*n* = 4–6). Data were thoroughly counted and analyzed using a *t*-test. **b** Enumeration of eggs in liver section (2 mm) from mice subjected to 6 weeks of *S. japonicum* infection, with or without ART or ATT treatment, was conducted and analyzed by *t*-test. Representative liver section (2 mm or 400 μm) post- H&E staining from these groups are exhibited in **c**. **d** Scanning electron microscopy (SEM) was employed to examine the surface morphology of adult worms extracted from the livers of infected mice, treated with or without ATT. Detailed views include the tegument of adult worms' mid-body without (a1) or with (a2) ATT treatment. ATT’s impact is evident in the altered appearance of regular prominent spines (b1 versus b2), crests (c1 versus c2) in the mid-body tegument and prominent spines (e1 versus e2) on the gynecophoral canal of adult males (d1 versus d2). **e**, **f** Volcano plots illustrate the differential expression of genes (DEGs) in *S. japonicum* female (**e**) and male (**f**) adult worms from mice with or without ATT treatment, as analyzed by RNA-seq analysis (FDR ≤ 0.001 and log_2_ ≥ 1). The *X* axis represents the log_2_ fold change, and the *Y* axis represents the log_10_ (adjusted *P* value). In these plots, red and green represent up- and downregulated genes, respectively, while gray dots denote genes without significant changes. The threshold was set at adjusted *P* value < 0.05 and |log_2_ fold change| > 1. **g**, **h** Significant Gene Ontology (GO) categories for DEGs in *S. japonicum* female (**h**) and male (**i**) adult worms from mice treated with or without ATT. The *X* axis denotes the log_10_ (*P* value), and the *Y* axis lists the GO term names

ART and its derivatives are known to decrease the number of female adult worms or increase the count of single male worms, thereby reducing the total burden of adult worms. Hererin, treatment with either ATT or ART with a very low dose (5 mg/kg body weight) significantly reduced the total number of *S. japonicum* adult worms in the portal and mesenteric veins of mice with schistosomiasis japonica, compared with the untreated group (Fig. 2a; *t*-test: *Sj*6w/NC versus *Sj*6w/ATT: *t*(6) = 2.43, *P* = 0.026). Notably, the count of single male worms increased significantly (Fig. 2a; *t*-test: *Sj*6w/NC versus *Sj*6w/ATT: *t*(8) = 2.53, *P* = 0.018. *Sj*6w/NC versus *Sj*6w/ART: single male: *t*(7) = 2.33, *P* = 0.026). Notably, the count of single male worms increased significantly (Fig. 2a). Furthermore, within the liver, ATT treatment significantly reduced the number of deposited eggs, a result not observed with ART (Fig. 2b, c; *t*-test: *Sj*6w/NC versus *Sj*6w/ATT: *t*(8) = 2.07, *P* = 0.036).

To further elucidate the schistosomicidal efficacy of ATT, adult female and male worms extracted from mice were examined using scanning electron microscopy (SEM). As shown in Fig. 2d, apparent alterations were observed in both the mid-body surface of adult females and males, as well as in the surface of the males’ gynecophoral canal. ATT treatment resulted in the loss of apically directed spines and rupture of the crests. Additionally, the tegument of the gynecophoral canal exhibited extensive damage characterized by structural disorganization, collapse, and the presence of hole-shaped erosions.

To further examine the possible molecular mechanism of ATT’s anti-worm effect, DEGs in adult female and male worms, isolated from *S. japonicum*-infected mice with or without ATT treatment, were analyzed through RNA-seq. Each sample yielded approximately 20 million total sequence reads. RNA-seq analysis revealed significant changes in the transcription of 98 genes in females (Fig. 2e) and 48 genes in males (Fig. 2f) due to ATT treatment. Gene Ontology (GO) enrichment analysis highlighted that GO terms such as “cellular anatomical entity,” “catalytic activity,” “binding,” and “cellular process” were significantly represented among these altered genes. Notably, the majority of genes were predominantly associated with “cellular anatomical entity” (Fig. 2g, h).

These results suggest that ATT directly damages the surface structure of *S. japonicum,* particularly the surface of males’ gynecophoral canal, contributing to the reduction of female worms (or the increase in single male worms) and, subsequently egg reduction. This is likely due to transcriptional changes in genes associated with cellular anatomical entities.

### Comprehensive analysis using both transcriptome and 23 cytokines’ assay of mouse liver lysates underscored the immune-modulatory effects of ATT

**Fig. 3:**
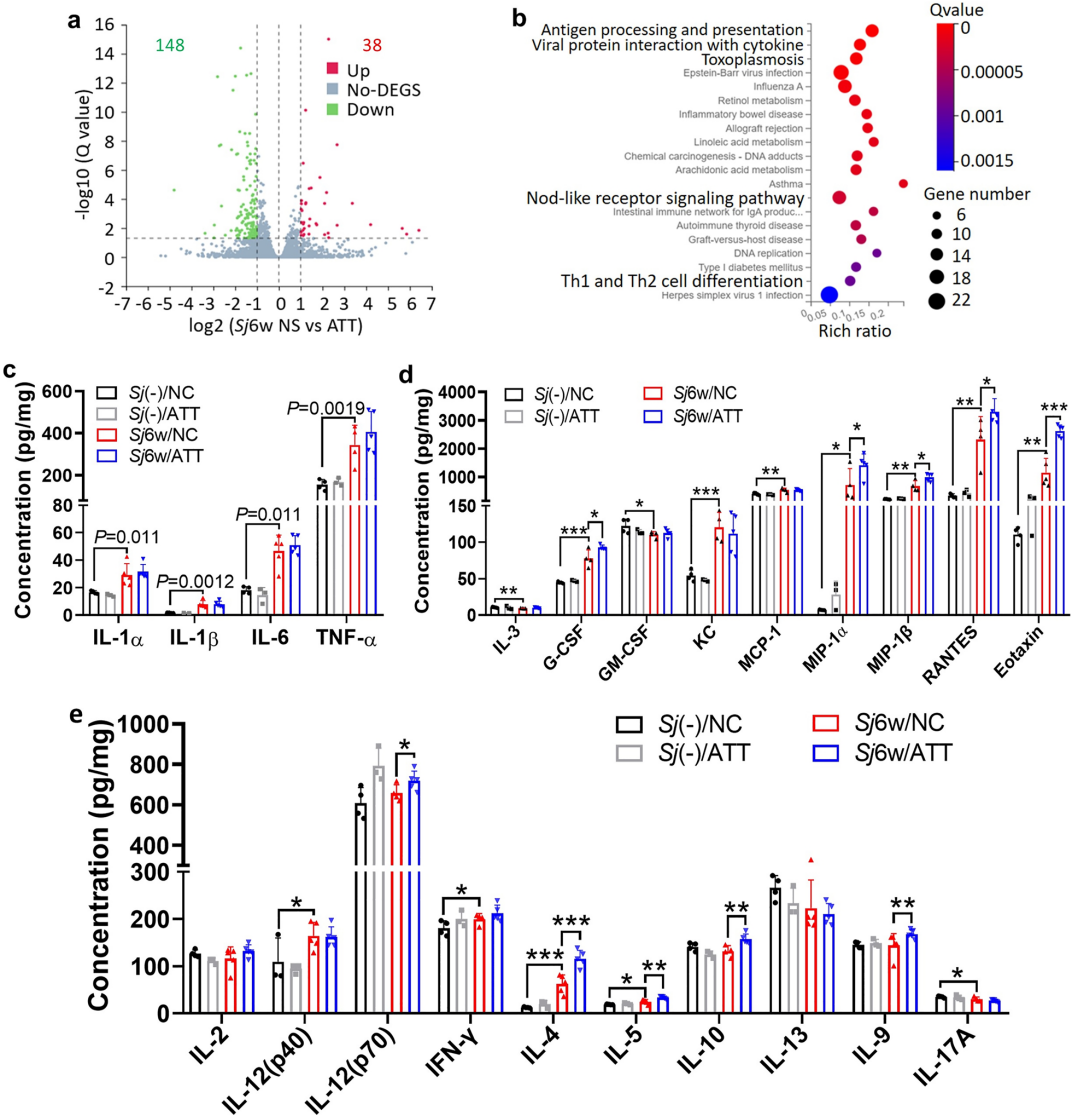
RNA-seq and multi-cytokine analysis indicated the immunomodulatory effects of ATT on the liver of mice with 6 weeks of *S. japonicum* infection. **a** Volcano plot illustrating the DEGs in *S. japonicum*-infected mice liver, with or without ATT treatment. The *X* axis represents the log_2_ fold change, while the *Y* axis shows the log_10_ (adjusted *P* value). In this plot, red and green symbols highlight upregulated and downregulated genes, respectively, while gray symbols indicate genes with no significant difference. The threshold for significance is a set at an adjusted *P* value < 0.05 and |log_2_ fold change| > 1; **b** Kyoto Encyclopedia of Genes and Genomes (KEGG) pathway enrichment analysis identifies significant pathways affected by DEGs in the infected mice liver, with or without ATT treatment. The *X* axis indicates −log_10_ (*P* value), and the *Y* axis lists the names of affected pathways. **c**–**e** Concentrations of 23 cytokines were measured using Bio-Plex Pro-Mouse Group I Cytokine 23-plex assay. These cytokines include: pro-inflammatory cytokines (IL-1α, IL-1β, IL-6, TNF-α) (**c**), colony-stimulating cytokines or chemokines (IL-3, G-CSF, GM-CSF, KC, MCP-1, MIP-1α, MIP-1β, RANTES, Eotaxin) (**d**), and activatory and differentiating cytokines for innate lymphoid cells or T cell subsets (IL-2, IL-12 (p40), IL-12 (p70), IFN-γ, IL-4, IL-5, IL-10, IL-13, IL-9 and IL-17A (**e**). Data represent mean ± SD from different experimental groups and were analyzed by *t*-test (*n* = 4–6). Significant differences are indicated by *P < 0.05 and **P < 0.01

To explore the mechanisms underlying ATT's influence on liver inflammation and fibrosis caused by 6 weeks of *S. japonicum* infection, RNA-seq and Bio-Plex Pro-Mouse Group I Cytokine 23-plex test were employed. RNA-seq revealed that ATT treatment significantly upregulated 38 genes and downregulated 148 genes in the liver tissues of infected mice (Fig. 3a). The Kyoto Encyclopedia of Gene and Genome (KEGG) pathway analysis identified that most of the genes related to immunomodulatory activities were enriched, including pathways like antigen processing and presentation, viral protein interaction with cytokines, inflammatory bowel disease, NOD-like receptor signaling, and Th1 and Th2 cell differentiation pathways (Fig. 3b).

The Bio-Plex Pro-Mouse Group I Cytokine 23-plex assay displayed significant changes in level of 18 cytokines and chemokines following 6 weeks of *S. japonicum* infection. There was a notable increase in proinflammatory cytokines such as IL-1α, IL-1β, IL-6, TNF-α (Fig. 3c; *t*-test: *Sj*(−)/NC versus *Sj*6w/NC: IL-1α: *t*(7) = 2.94, *P* = 0.011; IL-1β: *t*(7) = 4.60, *P* = 0.0012; IL-6: *t*(7) = 4.73, *P* = 0.0011; TNF-α: *t*(6) = 3.09, *P* = 0.0019), as well as colony-stimulating cytokines or chemokines such as G-CSF, KC, MCP-1, MIP-1α, MIP-1β, RANTES and Eotaxin (Fig. 3d; *t*-test: *Sj*(−)/NC versus *Sj*6w/NC: IL-3: *t*(6) = 3.349, *P* = 0.0077; G-CSF: *t*(6) = 5.49, *P* = 0.0008; GM-CSF: *t*(6) = 2.22, *P* = 0.034; KC: *t*(6) = 6.05, *P* = 0.0005; MCP-1: *t*(7) = 3.89, *P* = 0.0030; MIP-1α: *t*(6) = 2.47, *P* = 0.024; MIP-1β: *t*(6) = 4.881, *P* = 0.0014; RANTES: *t*(6) = 4.85, *P* = 0.0014; Eotaxin: *t*(7) = 4.05, *P* = 0.0024). Additionally, activator or differential cytokines for innate lymphoid cells or T cell subsets such as IL-12(p40), IFN-γ, IL-4, and IL-5 were significantly increased (Fig. 3e). Conversely, IL-3, GM-CSF and IL-17A levels were notably decreased. No significant changes were observed in IL-2, IL-10, IL-13, and IL-9 (Fig. 3e; *t*-test: *Sj*(−)/NC versus *Sj*6w/NC: IL-12(P40): *t*(6) = 2.003, *P* = 0.046; IFN-γ: *t*(6) = 1.98, *P* = 0.047; IL-4: *t*(7) = 4.99, *P* = 0.0008; IL-5: *t*(7) = 2.53, *P* = 0.019; IL-17A: *t*(6) = 1.98, *P* = 0.047). Post ATT treatment, there was a significant increase in G-CSF, MIP-1α, MIP-1β, RANTES, eotaxin (Fig. 3d; *t*-test: *Sj*6w/NC versus *Sj*6w/ATT: G-CSF: *t*(6) = 2.45, *P* = 0.025; MIP-1α: *t*(7) = 2.13, *P* = 0.035; MIP-1β: *t*(7) = 2.89, *P* = 0.012; RANTES: *t*(7) = 2.31, *P* = 0.027; eotaxin: *t*(8) = 5.97, *P* = 0.0002) and IL-12(p70), IL-4, IL-5, IL-10, and IL-9 (Fig. 3e; *t*-test: *Sj*6w/NC versus *Sj*6w/ATT: IL-12(P70): *t*(7) = 1.99, *P* = 0.043; IL-4: *t*(8) = 4.56, *P* = 0.0009; IL-5: *t*(8) = 3.91, *P* = 0.0022; IL-10: *t*(7) = 3.44, *P* = 0.0054; IL-9: *t*(8) = 2.03, *P* = 0.038) in the livers of infected mice. However, levels of IL-1α, IL-1β, IL-6, TNF-α (Fig. 3c), IL-3, GM-CSF, KC (Fig. 3d), IL-12(p40), IFN-γ, IL-13, and IL-17A (Fig. 3e) remained unchanged. These results suggest that ATT has significant immunomodulatory effects, especially in enhancing type 2 immune responses.

### Effect of ATT or ART treatment on the count of immune cells in the liver or spleen of mice with 6w of *S. japonicum* infection

**Fig. 4:**
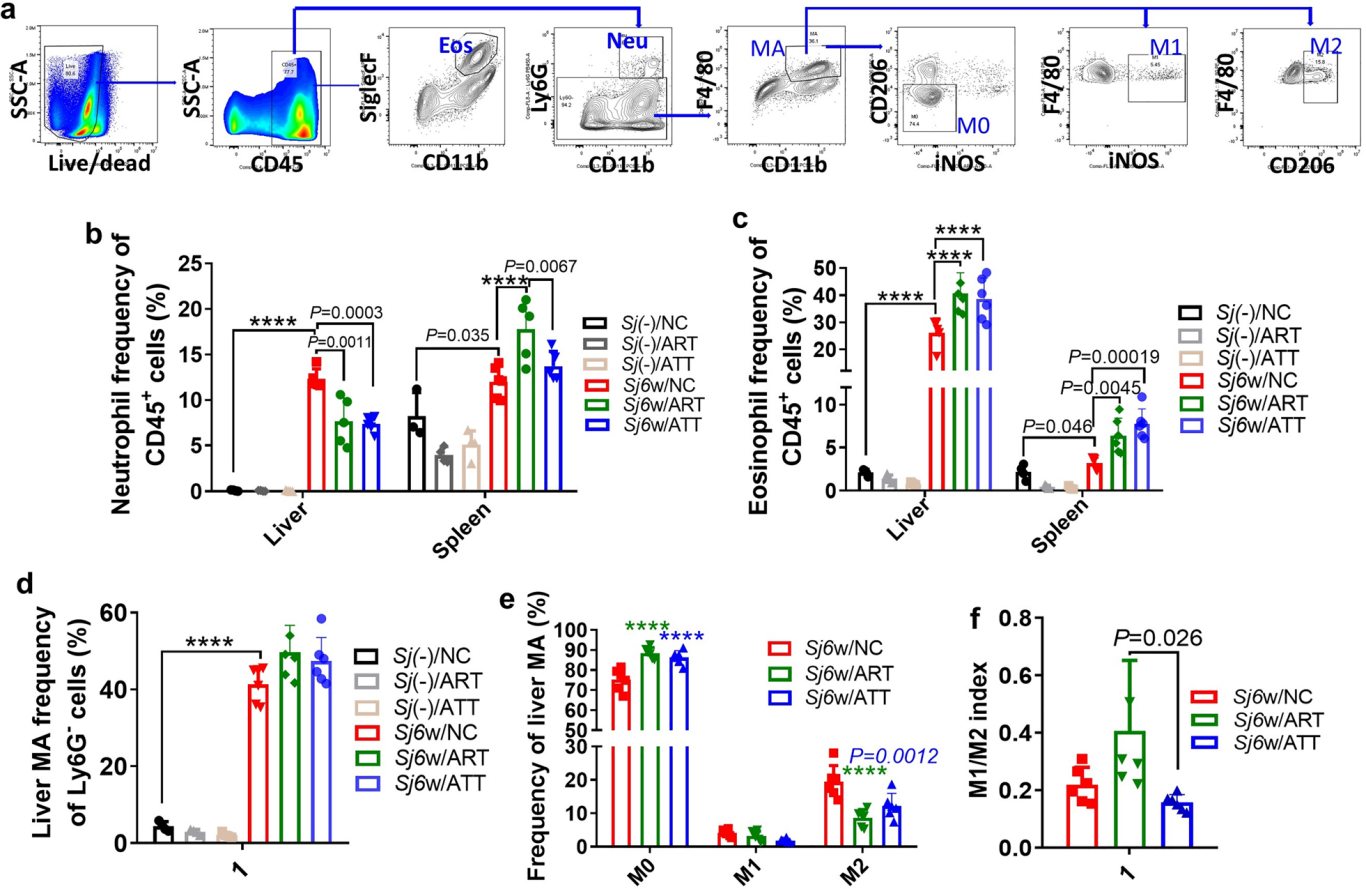
Neutrophil, eosinophil and macrophage redistribution in the liver or spleen of *S. japonicum*-infected mice following ART or ATT treatment. Flow cytometry was used for these cells analysis. Nucleated cells were initially gated on the basis of size and complexity, as indicated by forward scatter area (FSC-A) and side scatter area (SSC-A). Subsequently, single cells were distinguished from doublets using a combination of FSC-A and FSC-H. **a** Within this single cell population, live immune cells were identified as CD45^+^. Neutrophils (Neu) were further defined using CD45^+^CD11b^+^ Ly6G^+^ markers, and eosinophils (Eos) were gated as CD45^+^CD11b^+^SiglecF^+^. Macrophages (MA) were identified as CD45^+^Ly6G^−^F4/80^+^. These MA were further classified into M0 (CD206^−^iNOS^−^), M1 (iNOS^+^), and M2 (CD206^+^) subpopulations. **b** The proportion of neutrophils within CD45^+^ cell population in both liver and spleen of infected mice, with or without ART or ATT treatment, was evaluated and analyzed by two-way ANOVA (*n* = 4–6). **c** The proportion of eosinophils among CD45^+^ cells in both the liver and spleen of infected mice, treated with or without ART or ATT, was evaluated and analyzed by two-way ANOVA. **d**, **e** The proportions of total macrophages and their M0, M1, and M2 subpopulations in the CD45^+^Ly6G^−^ liver cells of infected mice, treated with or without ART or ATT, were evaluated and analyzed by one-way or two-way ANOVA, respectively. **f** The M1/M2 ratio (index) was presented and analyzed by one-way ANOVA. Data represent mean ± SD from different experimental groups. Significant differences are denoted by *P < 0.05, **P < 0.01, ***P < 0.001 and ****P < 0.0001

**Fig. 5:**
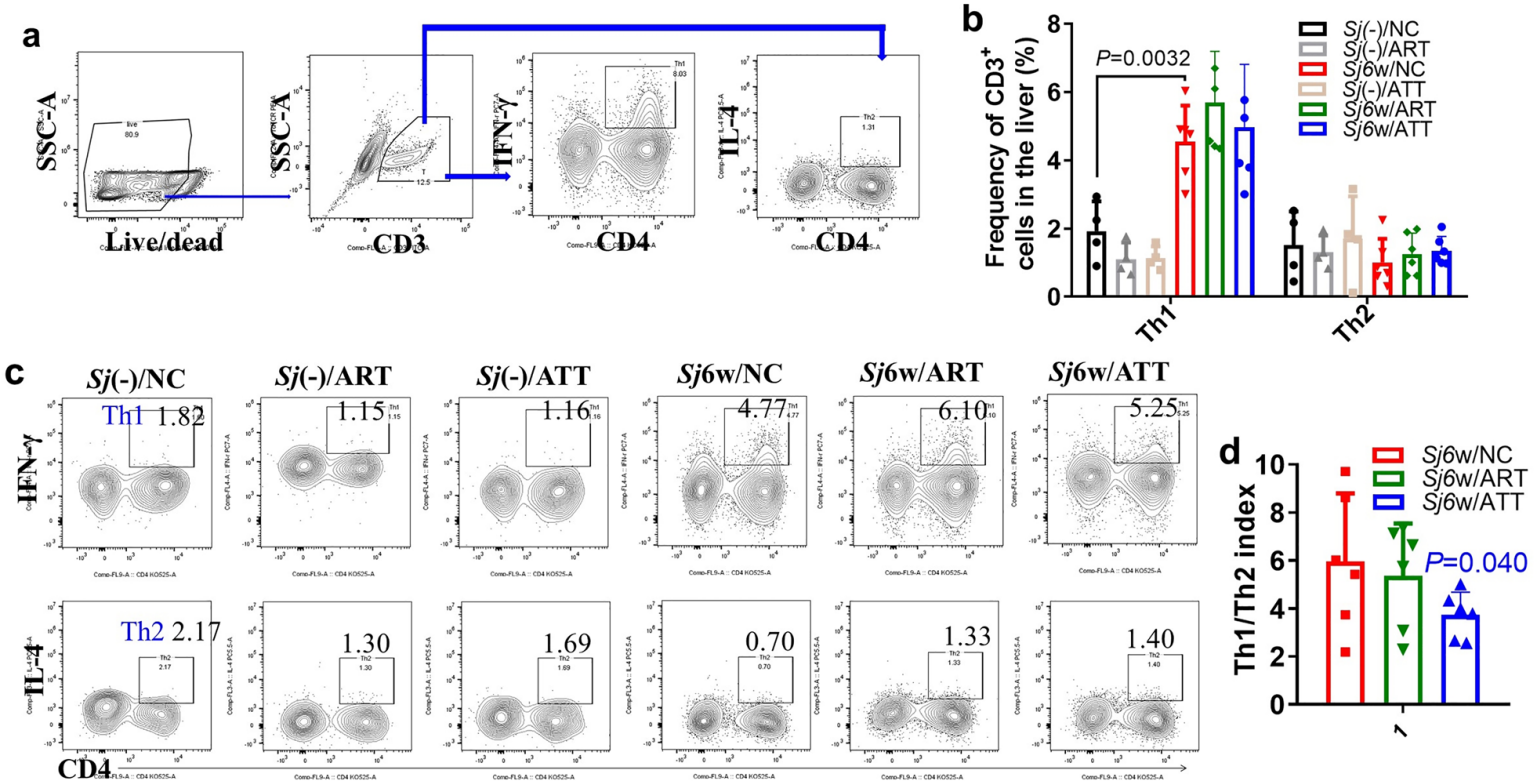
Altered Th1/Th2 index in the livers of infected mice treated with ATT compared with ART. **a** Flow cytometry was used to assess the proportion of Th1 and Th2 cells in the liver of indicated mice. The gating strategy involved identifying CD45^+^CD3^+^ cells, then subdividing them into CD45^+^CD3^+^CD4^+^IFN-γ^+^ (Th1) and IL-4^+^ (Th2) populations. **b**, **c** The amount of Th1 and Th2 cells in the liver of infected mice, with or without ART or ATT treatment, were evaluated. **a** and **b** display the typical cell populations for Th1 and Th2, and panel (**b**) providing their quantification, which was analyzed by two-way ANOVA. **d** The Th1/Th2 ratio (index) was analyzed by *t*-test. Data represent mean ± SD from different experimental groups. Significant differences are indicated by *P < 0.05 and **P < 0.01

Artemisinin analogs have demonstrated context-dependent immunomodulatory effects in various cell types and diseases, influencing both innate and adaptive immune responses. To explore the influence of ATT on immune cell populations in the liver and spleen of mice infected with *S. japonicum* for 6 weeks, mononuclear cells were isolated and analyzed using respective markers (Figs. 4a, 5a).

After a 6-week period of *S. japonicum* infection, there was a notably increase in the percentage of neutrophils in the liver and spleen. The increase in the liver was significantly reversed following treatment with ATT and ART. However, both ATT and ART significantly increased these levels in the spleen, with ART showing a particularly strong effect (Fig. 4b; ANOVA: *F*(5,43) = 11.31. *Sj*(−)/NC versus *Sj*6w/NC: liver: *P* < 0.0001; spleen: *P* = 0.035. *Sj*6w/NC versus *Sj*6w/ART: liver: *P* = 0.0011; spleen: *P* < 0.0001. *Sj*6w/NC versus *Sj*6w/ATT: liver: *P* = 0.0003; *Sj*6w/ART versus *Sj*6w/ATT: spleen: *P* = 0.0067). Regarding eosinophils, *S. japonicum* infection significantly increased their percentages. Further, both ATT and ART treatments significantly increased the frequency of eosinophils in the liver and spleen (Fig. 4c; ANOVA: *F*(5,46) = 40.06. *Sj*(−)/NC versus *Sj*6w/NC: liver: *P* < 0.0001; spleen: *P* = 0.046. *Sj*6w/NC versus *Sj*6w/ART: liver: *P* < 0.0001; spleen: *P* = 0.0045. *Sj*6w/NC versus *Sj*6w/ATT: liver: *P* < 0.0001; spleen: *P* = 0.00019). *S. japonicum* infection also significantly increased the number of macrophages in the liver. This increase was further accentuated but not significantly with the administration of either ATT or ART (Fig. 4d; ANOVA: *F*(5,24) = 118.8. *Sj*(−)/NC versus *Sj*6w/NC: *P* < 0.0001). The amount of M0 macrophages significantly increased, while the count of M2 macrophages substantially decreased with both ATT and ART treatments. The count of M1 macrophages was not significantly affected by either ART or ATT (Fig. 4e; ANOVA: *F* (4,45) = 22.75. *Sj*6w/NC versus *Sj*6w/ART: M0: *P* < 0.0001; M2: *P* < 0.0001. *Sj*6w/NC versus *Sj*6w/ATT: M0: *P* < 0.0001; M2: *P* = 0.0012). Notably, compared with ART, ATT therapy markedly lowered the M1/M2 index levels (Fig. 4f; ANOVA: *F*(2,15) = 4.68. *Sj*6w/ART versus *Sj*6w/ATT: *P* = 0.026).

Furthermore, 6 weeks of *S. japonicum* infection significantly increased the amount of Th1 cells in the liver but did not affect Th2 cells. Neither ATT nor ART treatment significantly altered the count of Th1 and Th2 cells (Fig. 5b, c; ANOVA: *F*(5,48) = 11.33, *P* < 0.0001. Th1: *P* = 0.0032). However, ATT resulted in a significant decrease in the Th1/Th2 index (Fig. 5d; *t*-test: *Sj*6w/NC versus *Sj*6w/ATT: *t*(10) = 1.80, *P* = 0.040).

### ATT-regulated host immunity was more strongly correlated with the extent of liver fibrosis and the count of single males compared with ART

**Fig. 6:**
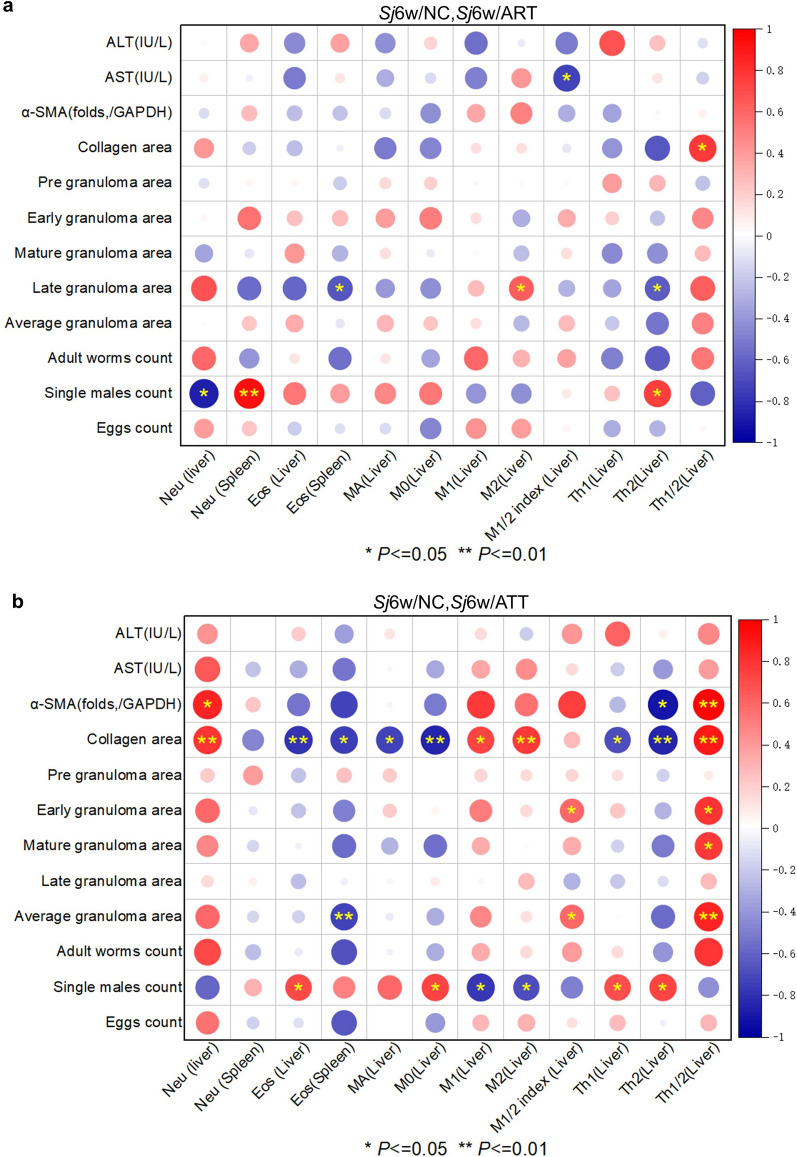
Correlation analysis of the frequency of host immune cells in the liver or spleen with the extent of liver inflammation or fibrosis, the size of granuloma area and the count of parasites. **a**, **b** Correlation plot of the frequency of neutrophils, eosinophils in the liver or spleen, the frequency of macrophages or their subsets M0, M1 or M2, M1/M2 index, and the frequency of Th1, Th2 and Th1/Th2 index in the liver with ALT and AST concentration, α-SMA expression folds (/GAPDH), area of collagen, pregranuloma, early granuloma, mature granuloma, late granuloma, and average granuloma, and count of worms, single males, and eggs from *S. japonica* at 6 weeks with or without ART (**a**) or ATT (**b**) treatment (*Sj*6w/NC, *Sj*6w/ART or *Sj*6W/ATT, *n* = 5–6) through OriginPro 2021. Significant correlation are indicated by *P < 0.05 and **P < 0.01

Correlation analysis highlighted more stars in 6 weeks of *S. japonicum-*-infected mice with ATT treatment than ART treatment (Fig. 6a, b). During ART treatment, significant negative correlations were observed between liver neutrophil frequency and single male counts, spleen eosinophil frequency and liver Th2 frequency with the area of late granulomas, and the M1/M2 index in the liver with serum AST concentration. Conversely, significant positive correlations were noted between spleen neutrophils frequency and liver Th2 frequency with single male counts, liver M2 frequency with the area of late granulomas, and the Th1/Th2 index with collagen area (Fig. 6a; correlation: AST and M1/2 index: *r*(10) = −0.53, *P* = 0.017; collagen area and Th1/2: *r*(10) = 0.58,* P* = 0.028; late granuloma area and Eos (Spleen): *r*(11) = −0.44, *P* = 0.038; late granuloma area and M2 (Liver): *r*(11) = 0.40, *P* = 0.048; late granuloma area and Th2 (Liver): *r*(11) = −0.41, *P* = 0.047; single males count and Neu (Liver): *r*(9) = −0.75, *P* = 0.012; single males count and Neu (spleen): *r*(9) = 0.88, *P* = 0.0018; single males count and Th2 (Liver): *r*(9) = 0.56, *P* = 0.021). During ATT treatment, significant negative correlations were observed between the frequency of Th2 in the liver and α-SMA folds, the frequency of Eos in the liver or spleen, and various immune cells, including MA, Mo, Th1, or Th2, with the collagen area. Additionally, a negative correlation was found between the frequency of Eos in the spleen and the average granuloma area, and between the frequency of M1 or M2 in the liver and the count of single males. Conversely, significant positive correlations were noted between the frequency of Neu, Th1/Th2 index in the liver and α-SMA folds or collagen area, the frequency of M1 or M2 with the collagen area, the M1/M2 or Th1/Th2 index in the liver with early or average granuloma areas, and the Th1/Th2 index with the area of mature granuloma. Additionally, positive correlations were found between the frequency of Eos, M0, Th1, or Th2 in the liver and the count of single males (Fig. 6b; correlation: α-SMA and Neu (Liver): *r*(6) = 0.73, *P* = 0.03; α-SMA and Th2 (Liver): *r*(6) = −0.83, *P* = 0.012; α-SMA and Th1/2: *r*(6) = 0.94,* P* = 0.0057; collagen area and Neu (liver): *r*(11) = 0.63, *P* = 0.0061; collagen area and Eos (liver): *r*(11) = −0.62,* P* = 0.0066; collagen area and Eos (spleen): *r*(11) = −0.56,* P* = 0.013; collagen area and MA (liver): *r*(11) = −0.54, *P* = 0.015; collagen area and M0 (liver): *r*(11) = −0.71, *P* = 0.0011; collagen area and M1 (liver): *r*(11) = 0.53, *P* = 0.017; collagen area and M2 (liver): *r*(11) = 0.59, *P* = 0.0094; collagen area and Th1 (liver): *r*(11) = −0.47, *P* = 0.028; collagen area and Th2 (liver): *r*(11) = −0.72,* P* = 0.0019; collagen area and Th1/2 (liver): *r*(11) = 0.78, *P* = 0.0034; early granuloma area and M1/2 index: *r*(12) = 0.34, *P* = 0.046; early granuloma area and Th1/2: *r*(12) = 0.61, *P* = 0.013; mature granuloma area and Th1/2: *r*(12) = 0.59, *P* = 0.015; average granuloma area and Eos (spleen): *r*(12) = −0.54,* P* = 0.0098; average granuloma area and M1/2 index: *r*(12) = 0.34, *P* = 0.045; average granuloma area and Th1/2: *r*(12) = 0.72,* P* = 0.0039; single males count and Eos (liver): *r*(10) = 0.49, *P* = 0.034; single males count and M0 (liver): *r*(10) = 0.54, *P* = 0.015; single males count and M1 (liver): *r*(10) = −0.61, *P* = 0.013; single males count and M2 (liver): *r*(10) = −0.47, *P* = 0.041; single males count and Th1 (Liver): *r*(10) = 0.49, *P* = 0.037; single males count and Th2 (liver): *r*(10) = 0.54, *P* = 0.015). ATT-regulated host immunity showed a more significant correlation with the extent of liver fibrosis and the count of single males compared with ART, suggesting that ATT is more effective than ART in treating murine schistosomiasis japonica.

## Discussion

In this study, we explored the comparative effects of ATT and ART on murine schistosomiasis japonica. Remarkably, ATT demonstrated superior anti-inflammatory, antifibrotic, and antischistosomal properties compared with ART. Mechanistically, ATT was found to injure the surface of *S. japonicum*, disrupting the tegument of adult worms, particularly the gynecophoral canal in males, which could affect male–female pairing and subsequent egg production. Both ART and ATT treatments reduced neutrophils but increased eosinophils and macrophages, especially M0 infiltration, into the mice’s liver. Unique to ATT is its ability to decrease the frequency of M1 and M1/M2 ratio, along with the Th1/Th2 indices, while increasing the concentration of type 2 cytokines IL-4, IL-5, and IL-10 in the liver. This host immune regulation by ATT correlates with significant reductions in liver fibrosis and the count of single male worms. These findings underscore ATT’s potential as a superior option over ART for preventing schistosomiasis japonica.

Some ART derivatives have been shown to be effective in treating malaria and schistosomiasis [17, 18, 20–23]. However, resistance to ART derivatives has emerged in *Plasmodium* strains [19]. ATT possesses a unique structural component, an α, β-unsaturated carbonyl group, which distinguishes it from ART. Prior to our study, the antiparasitic activity of ATT had not been explored. We provided the first solid evidence that the early-stage treatment (3–14 day post-*S. japonicum* infection) where ATT significantly combatted both the parasite and associated liver disease in mice with 6 weeks of *S. japonicum* infection. During this phase of ATT treatment, *S. japonicum* was developing into schistosomulum within the lungs and bloodstream. Notably, a low dose of ATT (5 mg/kg body weight) in the early stage demonstrated worm and egg reduction effects, which are not observed with praziquantel treatment. Furthermore, the lungs, stomach, and kidneys of mice treated with ATT displayed no abnormal appearance (data not shown). Therefore, ATT may have preventive efficacy against schistosomiasis japonica due to recent contact with *S. japonicum* cecariae-polluted water.

Scanning electron microscopy revealed that ATT treatment likely caused morphological injury to the teguments on the mid-body surface of adult worms and in the male gynecophoral canal. RNA-seq analysis revealed that ATT treatment significantly altered gene transcription in both male and female adult worms, with a notable enrichment in cellular anatomical entities. Further investigation is needed to confirm this effect, explore whether ATT impacts other internal structures of the parasites, and identify ATT’s targeting molecules or pathways inside *S. japonicum*. The tegument of adult worms is known to play a role in immune evasion [27], and the disruption caused by ATT could potentially enhance the efficacy of immune cells, particularly eosinophil-mediated immunity, against *S. japonicum* in the murine liver.

In mice infected with *S. japonicum* for 6 weeks, a typical liver granulomatous inflammation and fibrosis were observed, commonly utilized to assess treatment efficacy. The impact of ATT on liver disease and its potential immune regulation had not been previously documented. Our findings demonstrated that ATT effectively reduces liver inflammation and fibrosis. Through RNA-seq, multicytokines assay, and flow cytometry, we established that ATT enhances type 2 immunity, likely accounting for its antidisease efficacy. Although it contradicts reported studies that liver fibrosis is enhanced by type 2 immunity [28], liver granulomatous inflammation, rather than liver fibrosis, induced by 6 weeks of *S. japonicum* infection, was the major injury to the host liver. The immune-regulatory effect of ATT may be initiated by altered *S. japonicum* development, as ATT treatment alone [in the *Sj*(–)/ATT group] did not significantly change the baseline levels of cytokines or the frequency of immune cells, including neutrophils, eosinophils, macrophages, and Th1 and Th2 cells, in the liver. Conversely, the basal frequency of neutrophils and eosinophils in the spleen was considerably reduced by ATT or ART alone, consistent with the reported immunosuppressive effect of ART derivatives [29]. Although AST and ALT levels were significantly lowered by ATT in the sera of mice with 6 weeks of *S. japonicum* infection, the concentrations of IL-1α, IL-1β, IL-6, and TNF-α in the liver were not significantly decreased. These sustained high levels of proinflammatory cytokines might be crucial for maintaining the anti-parasitic effect, and could be a side effect of ATT.

In conclusion, this study presents compelling evidence that early-stage ATT treatment is significantly more effective against juvenile worms of *S. japonicum* compared with ART. This efficacy is attributed to ATT’s ability to disrupt the development of the tegument on the surface of *S. japonicum* and to enhance the host’s antiparasite immunity. Moreover, ATT exhibited stronger in vivo antidisease effects in the liver than ART, primarily through the amplification of type 2 immunity. Consequently, while this research highlights ATT’s potential as a preventive agent for schistosomiasis japonica, further investigation is essential to assess its targeting molecules or cells and its suitability for preventing this disease of populations with a history of recent contact with *S. japonicum* cecariae-polluted water. Further research is also needed to evaluate the therapeutic efficacy of ATT, including the optimal dosage, treatment timing, and duration.

## Data Availability

The data that support the findings of this study are available from the corresponding author or the first author upon reasonable request.
